# Introduction to Extreme Seeking Entropy

**DOI:** 10.3390/e22010093

**Published:** 2020-01-12

**Authors:** Jan Vrba, Jan Mareš

**Affiliations:** 1 Department of Computing and Control Engineering, Faculty of Chemical Engineering, University of Chemistry and Technology, 166 28 Prague, Czech Republic; 2 Department of Process Control, Faculty of Electrical Engineering and Informatics, University of Pardubice, 530 02 Pardubice, Czech Republic

**Keywords:** novelty detection, learning system, learning, time series, learning entropy, extreme seeking entropy

## Abstract

Recently, the concept of evaluating an unusually large learning effort of an adaptive system to detect novelties in the observed data was introduced. The present paper introduces a new measure of the learning effort of an adaptive system. The proposed method also uses adaptable parameters. Instead of a multi-scale enhanced approach, the generalized Pareto distribution is employed to estimate the probability of unusual updates, as well as for detecting novelties. This measure was successfully tested in various scenarios with (i) synthetic data, (ii) real time series datasets, and multiple adaptive filters and learning algorithms. The results of these experiments are presented.

## 1. Introduction

Novelty detection (ND) plays an important role in signal processing. Many research groups have dealt with both the methods and applications because there are many complex tasks where accurate ND is needed. However, the success of this method depends on the type of data, so the current methods usually give good performance and results only for specific datasets. As more data are being analyzed currently, there is a greater need for new methods of ND. Furthermore, the increasing computational power provides more possibilities and methods that were not possible to use a few decades ago, but can now be performed for real-time tasks easily. For these reasons, we consider the topic of ND to be vital.

Two different approaches have been established over the last few decades. The first approach is based on the statistical features of the data [1], and some methods also use extreme value theory to estimate the novelty of the data [2,3,4,5]. The second approach uses learning systems [6,7,8]: the attributes of a learning system are used to obtain information about novelties in the data. Over the last decade, many new methods have been proposed in the field of machine learning [9]. The set membership algorithm [10,11,12] uses the prediction error for better accuracy, reducing the computational resources required and assuring a greater robustness with the proper filter, especially for data without drift. Bukovsky et al. proved that the learning effort of a learning system can be used to estimate a measure of the novelty for each data point [13,14], but a shortcoming of that method is that it is hard to interpret the ND score. A similar approach, combining the prediction error with adaptive weight increments, was proposed in [15]. That method also lacks the possibility of a meaningful interpretation of the ND score. It was also already shown that the accuracy of the learning system is not necessarily correlated with the accuracy of the ND [16] and that simple predictors are useful even for signals that are produced by complex systems (e.g., EEG, ECG).

ND brings a new point of view to complex signal analysis. Research groups have started dealing with the early diagnosis of different diseases where ND plays an important role. Taoum et al. presented ND and data fusion methods to identify acute respiratory problems [17]. Rad introduced ND for gait and movement monitoring to diagnosis Parkinson’s disease and autism spectrum disorders [18]. Burlina used ND algorithms in the diagnosis of different muscle diseases [19].

Other fields where ND can be found are information and mechanical engineering. Hu introduced ND as an appropriate tool for monitoring the health of mechanical systems, where it is usually impossible to know every potential fault [20]. Surace described the application of ND to the simulation of an offshore steel platform [21].

In this article, a new method for ND is introduced. The proposed method combines both a statistics based approach and a learning systems based approach. The changes of the adaptive parameters of the learning system obtained via an incremental learning algorithm are evaluated. A new measure, called extreme seeking entropy, is then estimated. It is shown that the proposed measure corresponds to different types of novelties in various datasets and how it may be useful for diagnostics and failure detection tasks. It also outperforms the other unsupervised adaptive ND methods.

This paper is organized as follows. Section 2 describes the specifications of the learning system and learning algorithm used during the experiments. Section 3 recalls the learning entropy algorithm and an error and learning based novelty detection method. Then, the general suitable properties of learning based information are discussed. Section 4 introduces the new measure of novelty, and the ND algorithm based on this measure is presented. Section 5 describes a case study where both synthetic and real datasets are used to show the usability of the proposed algorithm and also contains the rationale behind the selection of the experiments. Section 6 contains the rate detection of the proposed algorithm in two cases, namely detection of a change in the trend and the detection of a step change of a signal generator. The last two sections are dedicated to limitations and further challenges, Section 7, and then our conclusions, Section 8.

## 2. Review of the Learning Systems Used

All the supervised learning systems used in the experimental analysis are introduced in this section. In general, assume that the output of the learning system is a function of weights and the input data:

(1) y=f(w,x)

where y∈R denotes the output, $w\in R^{n}$ is the vector of its adaptable parameters, $x\in R^{n}$ is a vector that contains the input data, and *f* is the mapping function that maps the input data and weights to the output. The following adaptation is done in order to minimize the error:

(2) e(k)=d(k)−y(k),

where *k* is a discrete time index and d(k)∈R is the target of the supervised learning system (the desired output). The update of the weights w is done with every new sample as follows:

(3) w(k+1)=w(k)+Δw(k)

where $\Delta w\in R^{n}$ is a vector that contains the updates of the adaptive parameters. This update depends on the learning algorithm used. The learning algorithms will be discussed later.

### 2.1. Adaptive Models

The adaptive models used during the experiments are described briefly in this section.

#### 2.1.1. Linear Adaptive Filter

One of the simplest adaptive models is the linear adaptive filter, also known as the linear neural unit (LNU), with finite impulse response (FIR). The output of this model at a discrete time index *k* is given by:

(4) $$y\left(k\right)=\sum_{i=1}^{n}w_{i}\cdot x_{i}\left(k\right),$$

which is equivalent to

(5) $$y\left(k\right)=w^{T}\left(k\right)\cdot x\left(k\right),$$

where $w^{T}\left(k\right)=\left[w_{1}\left(k\right),w_{2}\left(k\right),\ldots ,w_{n}\left(k\right)\right]\in R^{n}$ is the row vector of adaptive weights and $x^{T}\left(k\right)=\left[x_{1}\left(k\right),x_{2}\left(k\right),\ldots ,x_{n}\left(k\right)\right]\in R^{n}$ is the column input vector. The vector of adaptive weights is updated with every new sample obtained, and the size of the update depends on the learning algorithm used. In general, x may contain the history of a single input or even the history of multiple inputs.

#### 2.1.2. An Adaptive Filter Based on Higher Order Neural Units

The quadratic neural unit (QNU) [22,23,24] (also known as a second order neural unit) is a non-linear predictive model. The output of the QNU is:

(6) $$y\left(k\right)=\sum_{i=0}^{n}\sum_{j=i}^{n}w_{i,j}\cdot x_{i}\left(k\right)\cdot x_{j}\left(k\right)$$

where often, $x_{0}=1$. This is equivalent to:

(7) y(k)=w·colx,

where the column input vector colx for *n* inputs has the general form:

(8) $$\mathrm{colx}={\left[1,x_{1},\ldots ,x_{n},x_{1}^{2},x_{1}\cdot x_{2},\ldots ,x_{1}\cdot x_{n},x_{2}^{2},x_{2}\cdot x_{3},\ldots ,x_{2}\cdot x_{n},\ldots ,\ldots ,x_{n-1}\cdot x_{n},x_{n}^{2}\right]}^{T}$$

and w is a row vector of adaptive weights that has the same length as colx. Note that the first term in colx, $x_{0}=1$, should be used when the data have a non-zero offset.

### 2.2. Learning Algorithms

To prove the generality of the adaptive weight evaluation approach for novelty detection, different learning algorithms have been tested. Both algorithms are heavily used in signal processing and machine learning.

#### 2.2.1. Normalized Least Mean Squares Algorithm

The normalized least mean squares (NLMS) algorithm [25] is a variant of the least mean squares algorithm. The problem with the selection of the learning rate is solved by normalizing by the power of the input. It is a stochastic gradient approach. The update of this adaptive algorithm is given by:

(9) $$\Delta w\left(k\right)=\frac{\mu \cdot x(k)\cdot e(k)}{\epsilon +x^{T}\left(k\right)\cdot x\left(k\right)},$$

where ϵ∈R is a small positive constant used to avoid division by zero, μ∈R is the learning rate, and e∈R is the error defined as in (2). According to the normalization of the learning rate shown in (9), it is necessary to choose a learning rate μ satisfying 0≤μ≤2 to preserve the stability of the NLMS algorithm.

#### 2.2.2. Generalized Normalized Gradient Descent

The generalized normalized gradient descent (GNGD) [26] algorithm is another algorithm for linear adaptive FIR filters. Due to its adaptation of the learning rate based on the signal dynamics, it converges in places where the NLMS algorithm diverges. The update of this adaptive algorithm is given by:

(10) Δw(k)=η(k)e(k)x(k)

with:

$$\eta \left(k\right)=\frac{\mu }{{∥x(k)∥}_{2}^{2}+\epsilon \left(k\right)}$$

$$\epsilon \left(k\right)=\epsilon \left(k-1\right)-\rho \mu \frac{e\left(k\right)e\left(k-1\right)x^{T}\left(k\right)x\left(k-1\right)}{{\left({∥x(k-1)∥}_{2}^{2}+\epsilon \left(k-1\right)\right)}^{2}}$$

where η∈R is the adaptive learning rate, ϵ∈R is a compensation term, and ρ is the step size adaptation parameter, which should be chosen so as to satisfy 0≤ρ≤1.

## 3. On the Evaluation of the Increments in the Adaptive Weights in Order to Estimate the Novelty in the Data

This section recalls two ND methods that evaluate the increments in the adaptive weights, namely learning entropy, and error and learning based novelty detection. Those methods are compared with the proposed algorithm in Section 4 and Section 6. Then, the general properties of the learning based information measure will be discussed.

### 3.1. Learning Entropy: A Direct Algorithm

The recent publication on Learning Entropy [14] specifies a direct algorithm to estimate the learning entropy (LE) as follows.

(11) $$LE\left(k\right)=\sum_{i=1}^{n_{w}}z\left(\left|\Delta w_{i}\left(k\right)\right|\right)$$

Here, *z* is a special *Z*-score, given as follows:

(12) $$z\left(\left|\Delta w_{i}\left(k\right)\right|\right)=\frac{\left|\Delta w_{i}\left(k\right)\right|-\bar{\left|\Delta w_{i}^{M}\left(k-1\right)\right|}}{\sigma (\left|\Delta w_{i}^{M}\left(k-1\right)\right|)}$$

where $\bar{\left|\Delta w_{i}^{M}\left(k-1\right)\right|}$ is the mean of the last *M* increments of $w_{i}$, $\sigma (\left|\Delta w_{i}^{M}\left(k-1\right)\right|)$ is their standard deviation, and $n_{w}$ is the number of adaptive weights. According to Equation (15), the function *f* in this case corresponds to the special *Z*-score function *z*, and the function *A* is represented by the sum over the adaptive weights.

### 3.2. Error and Learning Based Novelty Detection

Another recently published method that evaluates the increments of the adaptive weight together with the prediction error is ELBND [15]. ELBND describes every sample with the value obtained as follows:

(13) $$ELBND\left(k\right)=\max_{1\leq i\leq n_{w}}\left|\Delta w_{i}\left(k\right)\cdot e\left(k\right)\right|$$

or, alternatively,

(14) $$ELBND\left(k\right)=\sum_{i=1}^{n_{w}}\left|\Delta w_{i}\left(k\right)\cdot e\left(k\right)\right|.$$

In this case, the function *f* is represented by multiplying the *i*th adaptive weight increment $\Delta w_{i}$ by the prediction error *e*. The function *A* is the maximum of the vector in the case of ELBND given by Equation (13) and the sum over the weights in the case of the ELBND given by Equation (14).

### 3.3. General Properties of A Suitable Learning Based Information Measure

Learning entropy was proposed in [13,14]. It is a learning based information measure *L* that, in general, evaluates unusually large learning increments, as follows:

(15) L(k)=A(f(Δw(k)))

where *A* is a general aggregation function and *f* is a function that quantifies the irregularity in the learning effort [14].

Another form for *f* and *A* will be presented in the present paper. Firstly, the function *f* is presented.

Assume that the value of *f* should be high when the increments Δw are unusually high. Furthermore, this function also takes the history of those increments as input. As stated, some cumulative distribution function of each weight increment seems suitable. This cumulative distribution function (cdf) is discussed later in this paper. The question is how to deal with the aggregation function *A*. Under the assumption that each weight is independent of the others, it is possible to choose the aggregation function *A* as follows:

(16) $$A(f\left(\Delta w\left(k\right)\right)=-\log\prod_{i=1}^{n}\left(1-f_{{cdf}_{i}}\left(\left|\Delta w_{i}\left(k\right)\right|\right)\right).$$

The function *A* in the stated form is high for high cdf values of the weight updates, and hence for the values where the cdf is close to one. The function $1-f_{{cdf}_{i}}$ can be viewed as the complementary cumulative distribution function (or the survival function, also known as the reliability function). This approach clearly avoids the need for a multi-scale approach. The result is that much fewer parameters are needed for detecting potential novelties. Only the crucial choice of the cdf remains. In the next section, a suitable probability distribution will be presented, together with the new novelty detection algorithm.

## 4. Extreme Seeking Entropy

### 4.1. The Generalized Pareto Distribution

A normal distribution is used in some novelty detection algorithms [27,28,29]. However, the normal distribution cannot always be used, especially when the description of the data by a mean and a symmetric range of variation would be misleading [30]. Let us mention the Pickands–Balkema–de Haan theorem [31,32], which states that if we have a sequence $X_{1},X_{2},\ldots$ of independent and identically distributed random variables and $F_{u}$ is their conditional excess distribution function (over the threshold *u*), then:

(17) $$F_{u}\left(x\right)\to GPD\left(\xi ,\mu ,\sigma \right)\left(x\right),\;\mathrm{as}\;u\to \infty$$

where GPD is the generalized Pareto distribution and $F_{u}$ is defined by:

(18) $$F_{u}\left(x\right)=P\left(X-u\leq x,X>u\right)=\frac{F(u+x)-F(x)}{1-F(x)}$$

for $0\leq x\leq x_{F}-u$, where $x_{F}$ is the right endpoint of the underlying unknown distribution *F*. The probability density function of the GPDtakes the form:

(19) $$f_{\left(\xi ,\mu ,\sigma \right)}\left(x\right)=\left\{\begin{array}{cc} \frac{1}{\sigma }{\left(1+\frac{\xi (x-\mu )}{\sigma }\right)}^{\left(-\frac{1}{\xi }-1\right)} & \mathrm{for}\;\xi \neq 0,\\ \exp\left(-\frac{x-\mu }{\sigma }\right) & \mathrm{for}\;\xi =0. \end{array}\right.$$

where in general, μ∈(−∞,+∞) is a location parameter, σ∈(0,∞) is the scale, and ξ∈(−∞,∞) is a shape parameter. The corresponding cumulative distribution function then takes the form:

(20) $$F_{\left(\xi ,\mu ,\sigma \right)}\left(x\right)=\left\{\begin{array}{cc} 1-{\left(1+\frac{\xi (x-\mu )}{\sigma }\right)}^{-\frac{1}{\xi }} & \mathrm{for}\;\xi \neq 0,\\ 1-\exp\left(-\frac{x-\mu }{\sigma }\right) & \mathrm{for}\;\xi =0. \end{array}\right.$$

**Figure 1:**
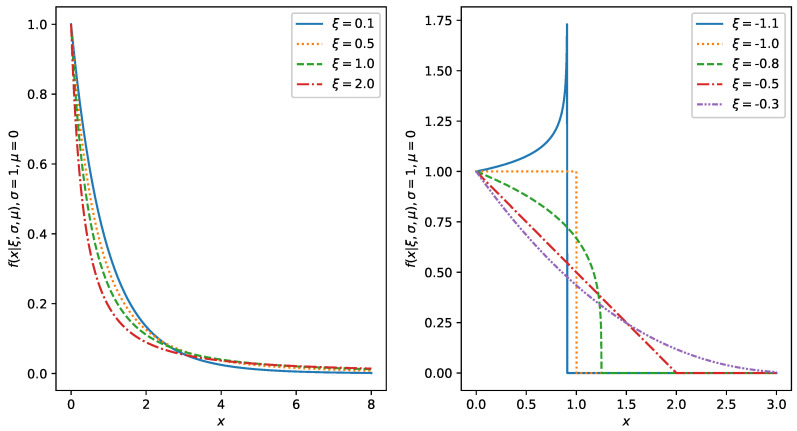
GPD probability density function with various parameters ξ, and fixed parameters σ=1, μ=0.

Note that the support is x≥μ if ξ≥0, and μ≤x≤μ−σ/ξ if ξ<0 where μ∈R, σ>0, and ξ∈R. In Figure 1, we show the ability of the GPD to deal with many possible shapes of the tails of the distributions. Note that if ξ=1, it is equivalent to the uniform distribution; if ξ=0, it is equivalent to the exponential distribution; if ξ=−0.5, it is the triangular distribution; if −0.5<ξ<0, it is a light tailed distribution (e.g., the normal distribution or the Gumbel distribution); if ξ>0, it is a heavy tailed distribution (e.g., the Pareto distribution, the log-normal distribution, or Student’s *t*-distribution); and if ξ<−1, it is a monotonically increasing distribution with compact support (e.g., the beta distribution).

As long as we do not know the distribution of increments of the adaptive weights, it is appropriate to use the GPD due to its universality in modeling the tails of other distributions [33,34,35]. As the aim is to evaluate unusually high increments of an adaptive system, the need for some threshold arises: denote this threshold by *z*. This threshold should divide the weight increments into two sets. An increment that is lower than the threshold should belong to the set that contains the usual high increments; denote this by *L*. However, an increment that is greater than or equal to this threshold should belong to the set *H*. Assume that both sets exist for every adaptable parameter, so for the *i*th adaptable parameter $w_{i}$, we should set a threshold $z_{i}$ so the weight updates belong to the sets as follows.

(21) $$\forall |\Delta w_{i}|<z_{i}\in L_{i}$$

(22) $$\forall |\Delta w_{i}|\geq z_{i}\in H_{i}$$

The increments belonging to $L_{i}$ will be unlikely to contain any information about a novelty in the adaptation, so we are not going to evaluate them. The set $H_{i}$ should contain the weight increments that are drawn from the GPD if the choice of the threshold was appropriate. The threshold $z_{i}$ depends on the method chosen, peaks over threshold, which will be discussed in the following subsection.

### 4.2. The Peaks over Threshold Method

The main issue in GPD fitting is the estimation of a suitable threshold, *z*. If the threshold is too high (i.e., there are only a few points that exceed it), then the parameters of the GPD suffer a high variance. If the threshold is too low, then the GPD approximation is not reliable. Therefore, the proper choice of threshold is crucial for the performance of the ND algorithm. There are many approaches to estimating the threshold [36]. To show the usability of the proposed ND algorithm, multiple rules of thumb [37,38,39] for the choice of the threshold have been used. Let *l* be the number of samples used for the GPD fitting and $n_{s}$ be the total number of samples available:

(23) $$l_{1}=⎡0.1\cdot n_{s}⎤$$

(24) $$l_{2}=⎡\sqrt{n_{s}}⎤$$

(25) $$l_{3}=⎡\frac{\sqrt[{3}]{n_{s}^{2}}}{log(log\left(n_{s}\right))}⎤$$

Note that we use the highest adaptive weight increment to estimate the GPD parameters. The peaks over threshold (POT) method is crucial for deciding whether $\left|\Delta w_{k}\left(k\right)\right|$ belongs to $H_{i}$ or to $L_{i}$. In Section 5 are presented the results with different techniques of choosing the threshold.

### 4.3. Extreme Seeking Entropy Algorithm

In this subsection, the new novelty measure and the new novelty detection algorithm are presented. We will introduce the extreme seeking entropy measure, which is given as follows:

(26) $$ESE\left(|\Delta w\left(k\right)|\right)=-\log\prod_{i=1}^{n}\left(1-f_{{cdf}_{i}}\left(\left|\Delta w_{i}\left(k\right)\right|\right)\right)$$

where:

(27) $$f_{cdf_{i}}(|\Delta w_{i}\left(k\right)|)=\left\{\begin{array}{c} 0,|\Delta w_{i}\left(k\right)|\in L_{i}\\ F_{\left(\xi_{i},\mu_{i},\sigma_{i}\right)}\left(|\Delta w_{i}\left(k\right)|\right),\left|\Delta w_{i}\left(k\right)\right|\in H_{i}. \end{array}\right.$$

The proposed algorithm evaluates the value of ESE for every newly obtained weight increment. Note that if the weight increment is smaller than the threshold from the POT method, the addition to the novelty measure ESE is zero. Small probability increments, which are highly likely to contain a novelty, have a high value of ESE. To estimate the parameters of the GPD pdf, it is possible to process all available history samples, or only the $n_{s}$ newest samples, with the POT method. The proposed algorithm is described by the following pseudocode (Algorithm 1).
**Algorithm 1** Extreme seeking entropy algorithm.1:set $n_{s}$, and choose the POT method2:initial estimation of the parameters of the GPD: $\xi_{i}$, $\mu_{i}$, $\sigma_{i}$ for each adaptable parameter3:**for** each new d(k) **do**4: update the adaptive model to get Δw(k)5: proceed with the POT method6: **if** $|\Delta w_{i}|\left(k\right)\in H_{i}$ **then**7:  update the parameters $\xi_{i}$, $\mu_{i}$, $\sigma_{i}$8: **end if**9: compute ESE according to (26)10:**end for**

The proposed ND algorithm needs only one parameter to be set, which avoids the need for a multi-scale approach and overcomes the issues arising from setting multiple parameters. The parameter $n_{s}$ can also take all available samples, if needed. Furthermore, there is the need to choose the proper POT method. Choosing the POT method depends strongly on the nature of the data. The limitation of the proposed method is the need for an initial estimate of the parameters of the GPD. We need a priori information about ξ, σ, and μ for each adaptive weight. If there are $n_{w}$ adaptive weights, then we need $3\cdot n_{w}$ parameters to start the extreme seeking entropy algorithm. If there is no a priori information about the parameters, we need at least $n_{s}$ samples to obtain the first results. Another problem may arise if the type of underlying unknown distribution *F* or its parameters are significantly varying in time.

## 5. Experimental Results

### 5.1. The Design of the Experiments

The proposed ESE algorithm was studied in various testing schemes with synthetic data and with one real dataset. For each experiment, we also show the results of the ELBND and LE methods, for the sake of comparison. The parameter *M* that specifies the number of increments for the LE evaluation was set as $M=n_{s}$ in all experiments. The first experiment was the detection of perturbed data in the Mackey–Glass time series. This experiment was chosen due to the possibility of comparing it with the results published in [13]. The second experiment, with synthetic data, showed the ability of the ESE algorithm to detect a change in the standard deviation of the noise in a random data stream, which can be viewed as a novelty in the data. It was inspired by a problem that arises in hybrid navigation systems that use both GPS and dead-reckoning sensors [40]. The third experiment, involving a step change in the parameters of a signal generator, was an analogue to a problem that may arise in evaluating multiple stream random number generators [41], where we may detect and evaluate the probability of changes in the parameters of those generators. The fourth experiment was the detection of the disappearance of noise. This experiment was chosen as neither of the compared methods (LE, ELBND) were able to deal with this problem, where the disappearance of the noise could be also viewed as a novelty in the signal. The fifth experiment was the detection of a change in trend; this is a common problem in fault detection and diagnosis [42]. The last experiment was performed on the mouse EEG dataset. The aim of this experiment was to show that the proposed ESE algorithm was suitable even for real-world complex phenomena that are characterized by non-linear dynamics [43,44]. This dataset contained the start of an epileptic seizure, and we wanted to show that it was possible to detect this seizure with the proposed ESE algorithm. All of the experiments were carried out in the programming language Python [45], with the libraries Numpy [46], Scipy [47], and Padasip [48]. The graphs were plotted with the Matplotlib library [49]. The codes with the experiments can be obtained via email from the authors.

### 5.2. Mackey–Glass Time Series Perturbation

The first experiment was the detection of a perturbed sample in a deterministic chaotic time series. The time series data were obtained as the solution of the Mackey–Glass equation [50].

(28) $$\frac{dy(t)}{dt}=\beta \cdot \frac{y(t-\tau )}{1+y^{\alpha }\left(t-\tau \right)}-\gamma y\left(t\right)$$

with parameters α=10, β=0.2, γ=0.1, and τ=17. In all, 701 data samples were generated. The data sample at discrete time index k=523 contained the perturbation, as follows:

(29) y(523)=y(523)+0.05·y(523)

**Figure 2:**
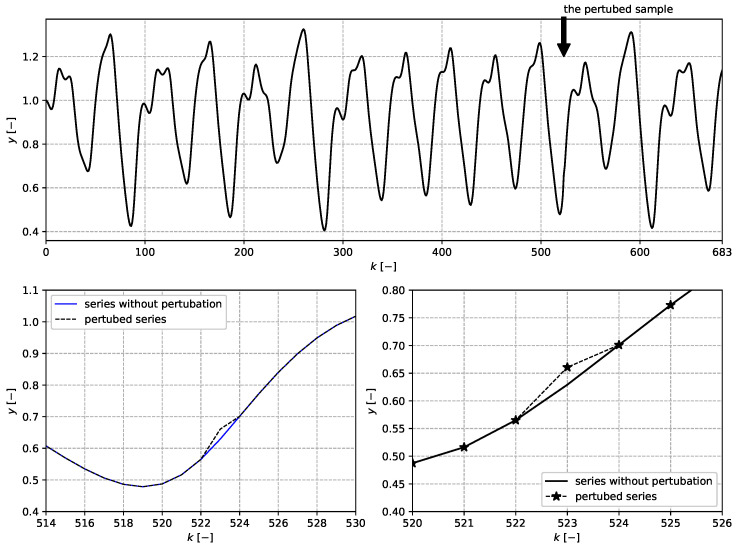
The (**upper**) plot displays an overview of the data with the perturbation. The (**bottom**) plots show the detailed perturbation.

The data series and detailed perturbation are depicted in Figure 2.

**Figure 3:**
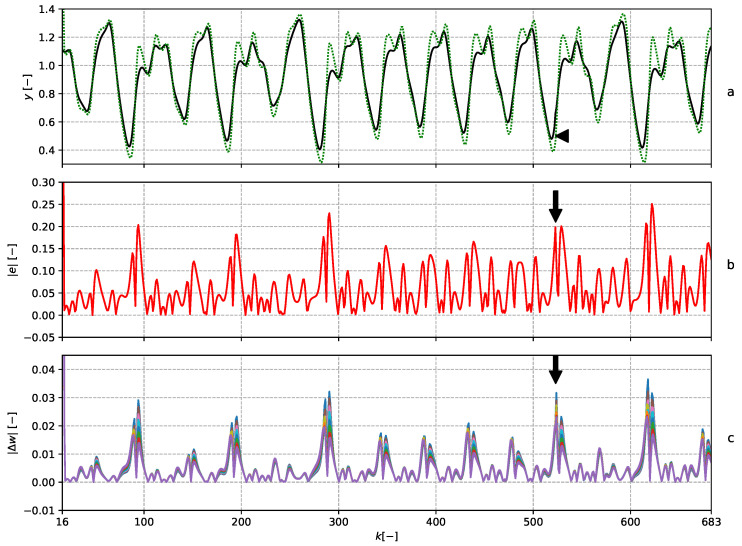
The graph (**a**) shows the data series with perturbation (solid black) and the output of the predictor (dotted green). The sample with perturbation is marked with the black arrow. The graph (**b**) shows the absolute value of the error of the predictor. The graph (**c**) shows the absolute value of the increments of the adaptable parameters.

**Figure 4:**
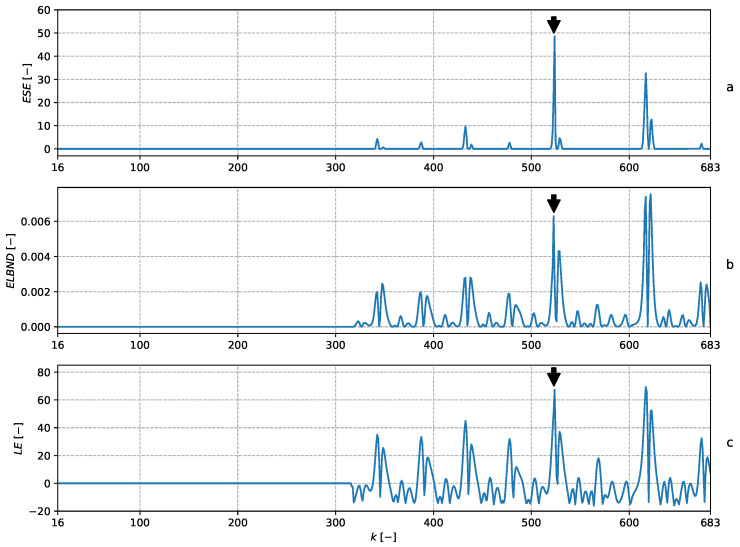
The graph (**a**) shows the ESE novelty score. Note that $n_{s}=300$, so the first 300 samples are needed to obtain the first value of ESE. The graph (**b**) shows the ELBND novelty score. The first 300 samples are set to zero for easier comparison with the other methods. The graph (**c**) shows the LE novelty score.

The QNU was chosen for the data processing. The number of inputs to the QNU was set to n=4, so the inputs are:

(30) x=[y(k−1),y(k−2),y(k−3),y(k−4)]

and hence, the adaptive filter had in all 15 adaptive weights. The parameters were updated with every newly obtained sample by means of the NLMS algorithm. The setting was the same as in [13]. The learning rate during the experiment was constantly set to μ=1. The POT method was chosen according to (23) with $n_{s}=300$. The details of the adaptive filters and prediction error are depicted in Figure 3. The results of the ND are shown in Figure 4. Note that the global maximum in the ESE corresponds to the perturbed sample. The global maxima of the ELBND and LE methods correspond to the biggest prediction error, and not to the perturbed sample.

### 5.3. Change of the Standard Deviation of the Noise in a Random Data Stream

**Figure 5:**
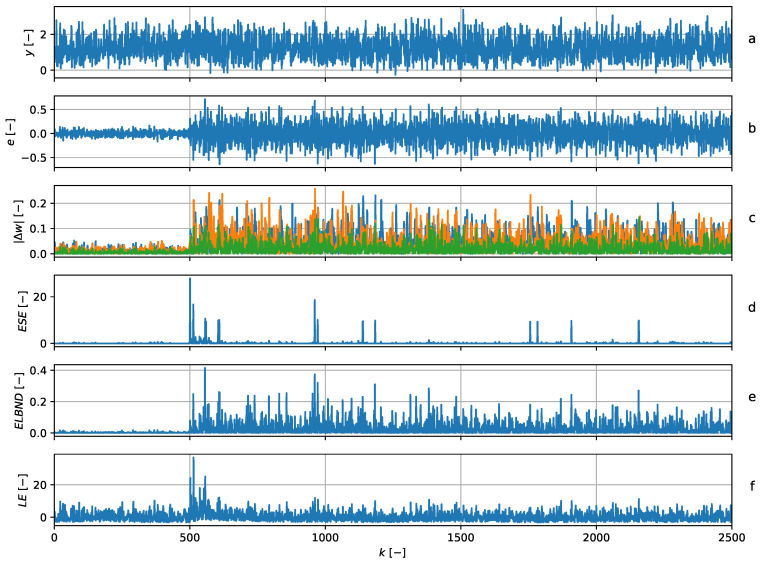
The graph (**a**) shows the data series (blue) and the output of the predictor (green). The graph (**b**) shows the error of the predictor. The graph (**c**) shows the absolute value of the increments of the adaptable parameters. The graph (**d**) shows the ESE novelty score. At discrete time index k=500, there is a step change in the standard deviation of the noise and a corresponding global peak in ESE. Graphs (**e**) and (**f**) contain the results of the ELBND and LE methods.

The detection of a change in the standard deviation of the noise in the obtained data was carried out in the following experiment. Assume there are two inputs $x_{1}\left(k\right)$ and $x_{2}\left(k\right)$ and that the output y(k) is related to them by:

(31) $$y\left(k\right)=x_{1}\left(k\right)+x_{2}\left(k\right)+x_{1}\left(k\right)\cdot x_{2}\left(k\right)+v\left(k\right)$$

where v(k) represents a Gaussian noise that is added to w(k). The Gaussian noise has zero mean and standard deviation 0.1, υ∼N(0,0.1). The values of $x_{1}\left(k\right)$ and $x_{2}\left(k\right)$ are drawn from a uniform distribution, so that x(k)≥0 and x(k)≤1 for every *k*. At the discrete time index k=500, the standard deviation of the noise changes to 0.2, so υ∼N(0,0.2). The QNU was chosen for the data processing. The number of inputs to the QNU was set to n=2, so the inputs are:

(32) $$x=[x_{1}\left(k-1\right),x_{2}\left(k-1\right)]$$

and hence, the adaptive filter had three adaptive weights in all. The structure of the QNU corresponds to the structure of the data generator described by Equation (31). The parameters were updated with every newly obtained sample using the GNGD algorithm. The learning rate during the experiment was set to μ=1. The POT method was chosen according to (24) with $n_{s}=500$. The results of the novelty detection and details about the adaptive filters are depicted in Figure 5. The a priori values of GPD for ESE and for LE were obtained using 500 samples, which are not shown in Figure 5. Note that the global maximum of the ESE corresponded to the change in standard deviation. The detection by the ELBND and LE was delayed.

### 5.4. Step Change in the Parameters of a Signal Generator

The scheme of this experiment was similar to the previous one. Assume there are two inputs $x_{1}\left(k\right)$ and $x_{2}\left(k\right)$ and one output y(k), related by:

(33) $$y\left(k\right)=x_{1}\left(k\right)+x_{2}\left(k\right)+x_{1}\left(k\right)\cdot x_{2}\left(k\right)+v\left(k\right)$$

where v(k) represents a Gaussian noise that is added to y(k). The Gaussian noise has zero mean and standard deviation 0.1, υ∼N(0,0.1). The values of $x_{1}\left(k\right)$ and $x_{2}\left(k\right)$ are drawn from a uniform distribution, so x(k)≥0 and x(k)≤1 for every *k*. At the discrete time index k=500, the equation is changed to the following one:

(34) $$y\left(k\right)=0.4x_{1}\left(k\right)+1.6x_{2}\left(k\right)+0.99x_{1}\left(k\right)\cdot x_{2}\left(k\right)+v\left(k\right).$$

**Figure 6:**
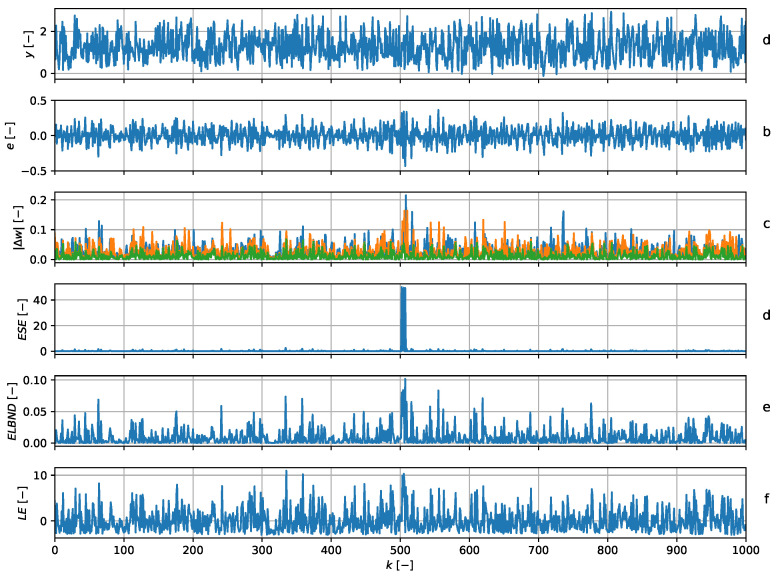
The graph (**a**) shows the data series (blue) and the output of the predictor (green). The graph (**b**) shows the value of predictors error. The graph (**c**) shows the absolute value of increments in the adaptable parameters. The graph (**d**) shows the ESE novelty score. At discrete time index k=500, there is a step change in the parameters and a corresponding global maximum in ESE. Graphs (**e**) and (**f**) contain the results of the ELBND and LE methods. The detection with ELBND is delayed. The LE method failed to detect the change in this case, as the global maximum of LE is at k=338.

The QNU was chosen for the data processing. The number of inputs to the QNU was set to n=2, so the inputs are:

(35) $$x=[x_{1}\left(k-1\right),x_{2}\left(k-1\right)]$$

and hence, the adaptive filter had three adaptive weights in all. Note that the structure of the QNU corresponded to the structure of the signal generator. The parameters were updated with every newly obtained sample, using the GNGD algorithm. The learning rate during the experiment was constantly set to μ=1. The POT method was chosen according to (23) with $n_{s}=500$. The a priori values of GPD for ESE and for LE were obtained using 500 samples, which are not shown in Figure 6. The results of the novelty detection and details about the adaptive filters are depicted in Figure 6. Note that the ESE successfully detected the change in the parameters of the signal generator. The LE failed to detect this change, and the detection by ELBND was delayed. Furthermore, the value of the peak in ESE was significantly higher than that in the ELBND case.

### 5.5. Noise Disappearance

In this experiment, it was shown that the slightly reformulated algorithm could also deal with an immediate decrease of the learning effort. Assume that instead of an unusually high learning effort, we want to focus on an unusually low learning effort. The only change in the proposed algorithm was that we used the POT method to get *l* the smallest weight updates, and based on those, the parameters of the GPD would be estimated. The scheme of this experiment was similar to the previous one. We assumed there were two inputs $x_{1}\left(k\right)$ and $x_{2}\left(k\right)$ and one output y(k), which were related by (31). However, in this case, at discrete time index k=500, the noise was removed, so Equation (31) for k≥500 takes the form:

(36) $$y\left(k\right)=x_{1}\left(k\right)+x_{2}\left(k\right)+x_{1}\left(k\right)x_{2}\left(k\right).$$

**Figure 7:**
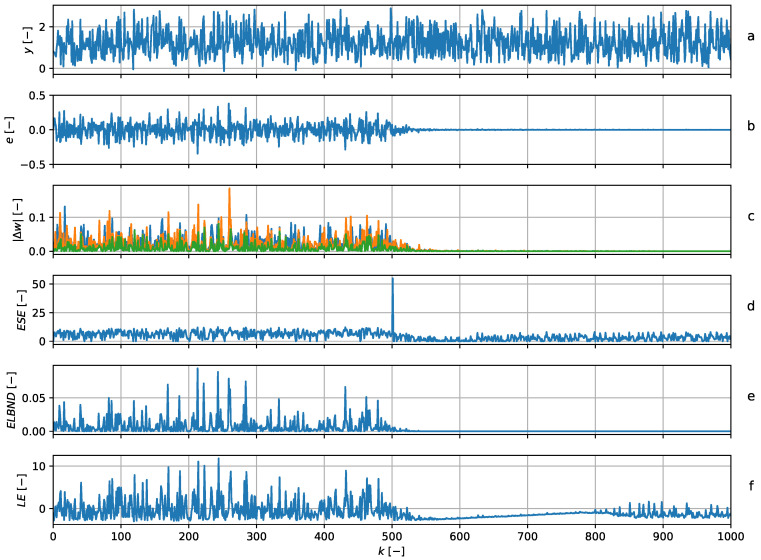
Noise disappearance detection. The graph (**a**) shows the data series (blue) and the output of the predictor (green). The graph (**b**) shows the error of the predictor. The graph (**c**) shows the absolute value of the increments of the adaptable parameters. The graph (**d**) shows the ESE novelty score. For the discrete time index k≥500, the noise is removed from the signal, which corresponds to the peak in ESE. Graphs (**e**) and (**f**) contain the results of the ELBND and LE methods.

The QNU was chosen for the data processing. The number of inputs to the QNU was set to n=2, so the inputs are:

(37) $$x=[x_{1}\left(k-1\right),x_{2}\left(k-1\right)]$$

and so, the adaptive filter had three adaptive weights in all. The structure of the adaptive filter was chosen to correspond to the structure of the signal generator. The parameters were updated with every newly obtained sample using the GNGD algorithm. The learning rate during the experiment was constantly set to μ=1. The POT method was chosen according to (23) with $n_{s}=500$. Figure 7 shows that the peak in ESE corresponded to the disappearance of the noise. The LE and ELBND methods failed to detect the disappearance of the noise. For ELBND, these results were to be expected, as the values of the ELBND were high for a high prediction error and high adaptive weight increments.

### 5.6. Trend Change

**Figure 8:**
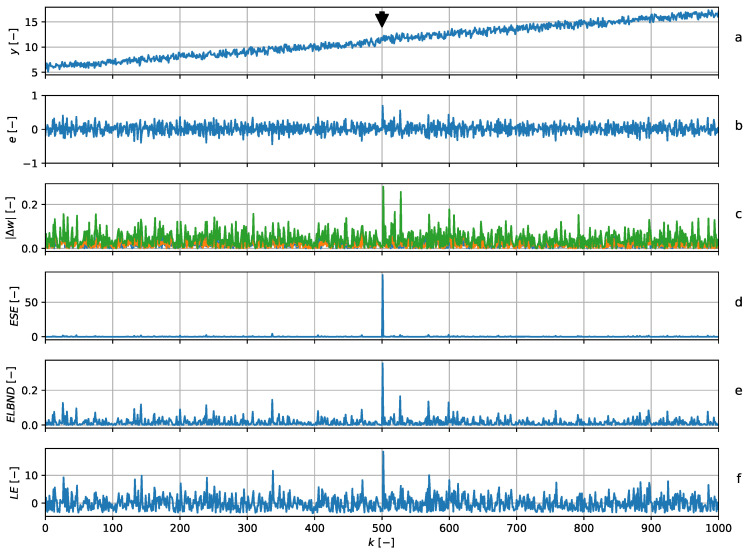
Trend change detection. The graph (**a**) shows the data series (blue) and the output of the predictor (green). The black arrow indicates the trend change. The graph (**b**) shows the error of the predictor. The graph (**c**) shows the absolute values of the increments in the adaptable parameters. The graph (**d**) shows the ESE novelty score. At discrete time index k=500, there is a step change in the trend, which corresponds to the peak in ESE. Graphs (**e**) and (**f**) contain the results of the ELBND and LE methods and peaks corresponding to the trend change.

The last experiment with artificial data was the detection of a change in trend. Assume that there are two inputs $x_{1}\left(k\right)$ and $x_{2}\left(k\right)$ and one output y(k), related by:

(38) $$y\left(k\right)=x_{1}\left(k\right)+x_{2}\left(k\right)+0.01\cdot k+v\left(k\right)$$

where v(k) represents a Gaussian noise that is added to y(k). The Gaussian noise had zero mean and standard deviation 0.1. At the discrete time index k=500, there was a change in the trend, so Equation (38) changes to:

(39) $$y\left(k\right)=x_{1}\left(k\right)+x_{2}\left(k\right)+0.0105\cdot k+v\left(k\right),$$

where k≥500. The LNU was chosen for the data processing. The number of inputs to the LNU was set to n=3, so the inputs are:

(40) $$x=[x_{1}\left(k-1\right),x_{2}\left(k-1\right),1]$$

and the adaptive filter had three adaptive weights in all. The structure of the adaptive filter was chosen in accordance with the structure of the signal generator. The parameters were updated with every newly obtained sample by means of the GNGD algorithm. The learning rate during the experiment was constantly set to μ=1. The POT method was chosen according to (23) with $n_{s}=500$. Figure 8 shows that the peak in ESE corresponded to the trend change point, which was the same as the peak in LE and ELBND. Note that the value of the peak in ESE was significantly higher than in LE and ELBND.

### 5.7. Detection of Epilepsy in Mouse EEG

**Figure 9:**
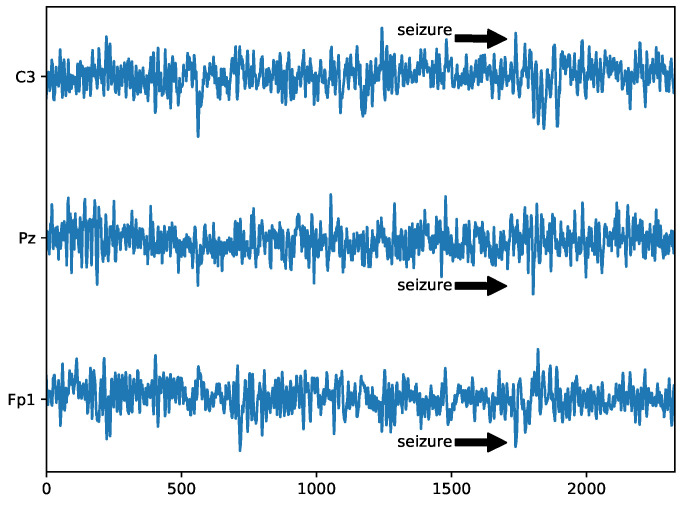
Selected mouse EEG channels with significant seizure. The data were standardized. The start of the seizure is approximately at discrete time index k≈1700.

The last experiment was with a mouse EEG signal. Three channels of the EEG data were chosen, which contained a significant seizure. According to the expert, the seizure started at about k≈1700, as is shown in Figure 9, which shows the *z*-scores of the EEG data.

**Figure 10:**
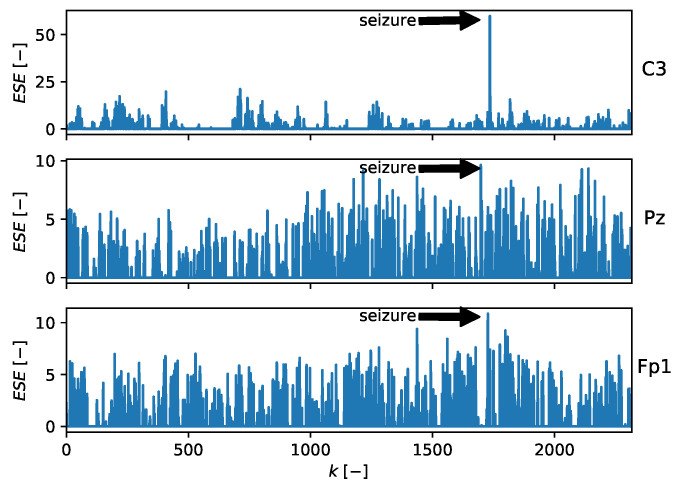
ESE value for mouse EEG data channels containing a seizure. The peaks approximately correspond to the beginning of the seizure. Note that channel C3 contains a significant peak in ESE compared to the other channels.

The LNU was chosen for the data processing. The number of inputs to LNU was set to n=10, so the inputs are:

(41) x=[x(k−1),x(k−2),⋯,x(k−10)]

and the adaptive filter had 10 adaptive weights in all. The number of inputs and filter structure were chosen experimentally. The parameters were updated with every newly obtained sample using the NLMS algorithm. The learning rate during the experiment was set to μ=1. The POT method was chosen according to (25) with $n_{s}=1000$. Figure 10 shows that the peak in ESE approximately corresponded to the beginning of the seizure. Especially in channel C3, the peak in ESE was significant. The position of the peaks was at k=1735 for channel C3, k=1698 for channel Pz, and k=1727 for channel Fp1.

## 6. Evaluation of the ESE Detection Rate

This section is dedicated to evaluating the detection rate in two different cases. The first case was a step change in the parameters of a signal generator (similar to the experiment described in Section 5.4). The second case was the detection of a change in trend.

### 6.1. Step Change in the Parameters of a Signal Generator: Evaluation of the Detection Rate

Assume there are two inputs $x_{1}\left(k\right)$ and $x_{2}\left(k\right)$, one output y(k), and weights $a_{1}$, $a_{2}$, and $a_{3}$, related by:

(42) $$y\left(k\right)=a_{1}\cdot x_{1}\left(k\right)+a_{2}\cdot x_{2}\left(k\right)+a_{3}\cdot x_{1}\left(k\right)\cdot x_{2}\left(k\right)+v\left(k\right)$$

where v(k) represents a Gaussian noise that is added to y(k). The Gaussian noise had zero mean and standard deviation σ. The initial values of $a_{1}$, $a_{2}$, $a_{3}$ were drawn from the uniform distribution U(−1,1). At discrete time index k=200, there was a step change in $a_{1}$, $a_{2}$, and $a_{3}$, and their new values were drawn again from U(−1,1). The structure of the adaptive filter was the same as described in Section 5.4. The parameters were updated with every newly obtained sample using the GNGD algorithm. The POT method was chosen according to (23) with $n_{s}=1200$. The performance of the ESE algorithm was compared with those of LE, ELBND, and plain prediction error evaluation. The a priori values of GPD for ESE and LE were obtained using 1200 samples with initial values for the parameters $a_{1}$, $a_{2}$, $a_{3}$. For each experiment, the signal-to-noise ratio (SNR) was evaluated as follows:

(43) $$SNR=10\log_{10}\frac{\sigma_{s}^{2}}{\sigma^{2}}$$

where $\sigma_{s}$ is the standard deviation of the output of the system and σ is the standard deviation of the noise. The evaluation of the rate detection was performed as follows:

1. choose noise standard deviation σ
2. for given noise standard deviation σ, perform 1000 experiments, and at the beginning of each experiment, choose new parameters $a_{1}$, $a_{2}$, and $a_{3}$
3. successful detection was when the global peak in ESE, LE, ELBND, or prediction error was between discrete time index k≥200 and k≤210; compute the detection rate
4. compute the SNR for each experiment according to (43), and compute the average SNR for all experiments for given noise standard deviation σ

**Figure 11:**
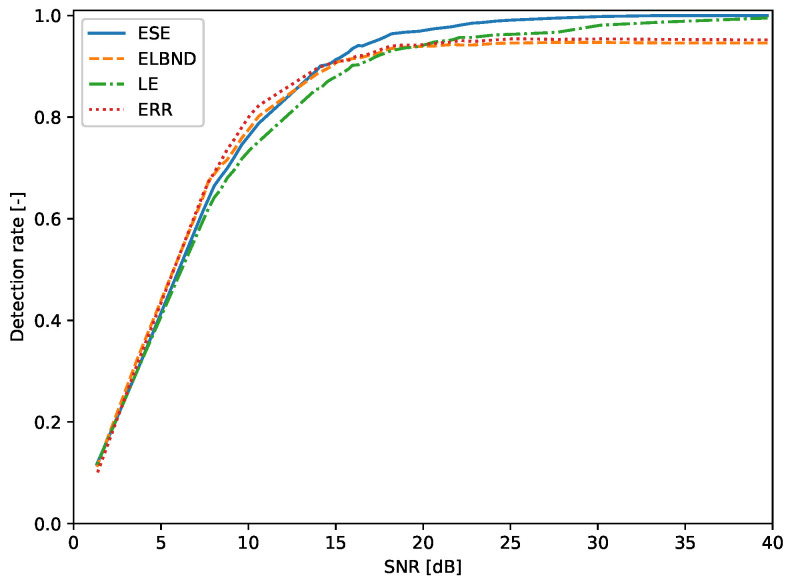
Detection of the step change in the parameters of a signal generator. The inputs of the signal generator are drawn from the uniform distribution U(−1,1). For SNR>15 dB, the ESE algorithm outperforms in the detection rate the LE, ELBND, and error evaluation. For SNR>33 dB, the ESE achieved a 100% detection rate.

**Figure 12:**
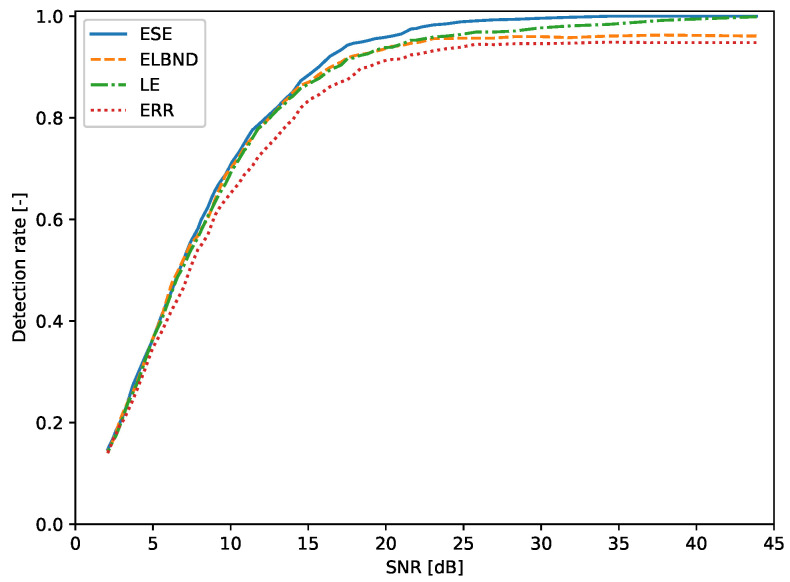
Detection of the step change in parameters of a signal generator. The inputs of the signal generator are drawn from the normal distribution N(0,1). For SNR>8 dB, the ESE algorithm outperforms in the detection rate the LE, ELBND, and error evaluation. For SNR>34 dB, the ESE achieved a 100% detection rate.

The evaluation of the detection rate was performed for the inputs $x_{1}$, $x_{2}$ whose values were drawn from the uniform distribution U(−1,1) and from the normal distribution N(0,1). The results for the inputs drawn from the uniform distribution are depicted in Figure 11. The corresponding table with results for various SNRs is Table A2 (see Appendix A). The results for inputs drawn from the normal distribution are depicted in Figure 12. The corresponding table with results for various SNRs is Table A3 (see Appendix A).

### 6.2. Detection of a Change in Trend: Evaluation of the Detection Rate

Assume there are two inputs $x_{1}\left(k\right)$ and $x_{2}\left(k\right)$ and one output y(k), related by:

(44) $$y\left(k\right)=x_{1}\left(k\right)+x_{2}\left(k\right)+0.01\cdot k+v\left(k\right)$$

where v(k) represents a Gaussian noise that is added to y(k). The Gaussian noise has zero mean and standard deviation σ. At discrete time index *k*, the trend changed, so the output of the system y(k) for k≥200 is given by:

(45) $$y\left(k\right)=x_{1}\left(k\right)+x_{2}\left(k\right)+\left(0.01+a\right)\cdot k+v\left(k\right)$$

where *a* is drawn from the uniform distribution U(−0.02,0.02). The structure of the adaptive filter was the same as in the experiment described in Section 5.6. The parameters were updated with every newly obtained sample using the GNGD algorithm. The POT method was chosen according to (23) with $n_{s}=1200$. The performance of the ESE algorithm was compared with LE, ELBND, and plain prediction error evaluation. The a priori values of the GPD for ESE and LE were obtained using 1200 samples where the output of the system was described by Equation (44). For each experiment, the SNR was evaluated according to (43). The evaluation of the rate detection was performed as follows:

1. choose noise standard deviation σ
2. for given noise standard deviation σ, perform 1000 experiments where at k=200, there is a change in trend
3. successful detection is when the global peak in ESE, LE, ELBND, or prediction error is between discrete time index k≥200 and k≤210; compute the detection rate
4. compute the SNR for each experiment according to (43), and compute the average SNR for all experiments for given noise standard deviation σ

**Figure 13:**
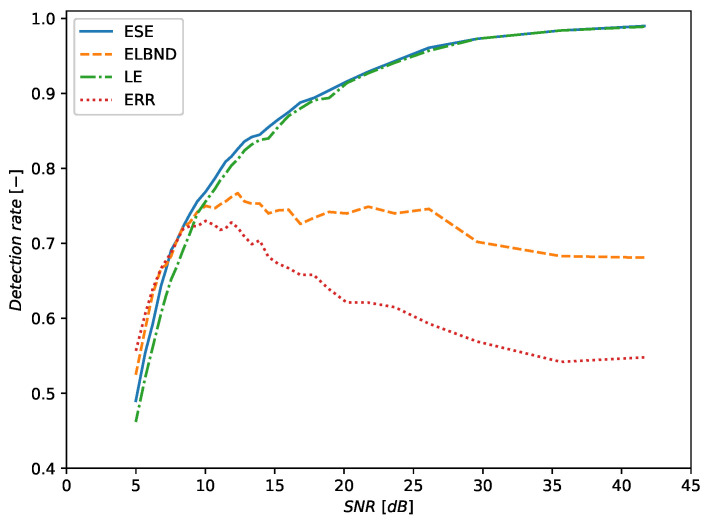
Detection of the trend change. The inputs of the signal generator are drawn from the uniform distribution U(−1,1). For SNR>8 dB, the ESE algorithm outperforms in the detection rate the LE, ELBND, and error evaluation.

The evaluation of the detection rate was performed for inputs $x_{1}$, $x_{2}$ whose values were drawn from the uniform distribution U(−1,1). The results are depicted in Figure 13. The corresponding table with the results for various SNRs is Table A1 (see Appendix A).

## 7. Limitations and Further Challenges

There is a significant limitation to using the ESE algorithm. As was already mentioned in Section 4, before we could obtain the first results, we needed to get a priori information about the parameters of the GPD or obtain a suitably large sample size to compute those parameters. This limitation arose from the nature of using the probability distribution and is common to many statistical approaches to ND. This was the main drawback compared to, e.g., the ELBND method, which was able to produce the results immediately. Another limitation of the presented algorithm is the selection of a suitable POT method, as the estimation of the parameters of the GPD and the selection of the threshold were strongly related to this. To avoid this issue, it was possible to implement some sophisticated parameter estimator that could deal with the optimal threshold selection (e.g., Zhang’s method [51], an estimator based on generalized probability weighted moment equations [52], or a method that combines the method of moments and the likelihood moment [53]), but these are outside the scope of this article. Another challenge was how to combine the ESE of unusually low and unusually high increments together, because both could correspond to a novelty in the data. Further work will be oriented toward using adaptive filters whose adaptive parameters are non-linearly related to the output, e.g., fuzzy adaptive filters or non-linear adaptive Kalman filters. Furthermore, more learning algorithms should be tested. Another topic, which was not mentioned in this article, is that of deciding whether the value of the ESE implies a novelty in the data or not, so we need some threshold. To evaluate the precision of the classification, the area under the receiver operating characteristics [54,55] should be estimated. Due to the scope of this article, this was omitted, but it will be part of further work on the ESE.

## 8. Conclusions

This paper introduced a new measure of data novelty, called extreme seeking entropy, and a detection algorithm that used this measure. An experimental study was also presented. The algorithm evaluated the absolute value of the increments in the adaptive system weights that were unusually high. The generalized Pareto distribution was used to model those increments, and we tested whether a low probability of a weight increment corresponded to a novelty in the data. It was also shown that the prediction error did not need to be correlated with a novelty in the data, so relatively simple, even inaccurate, adaptive models could be used. Five experiments with synthetic data including novelties and one experiment with a real mouse EEG signal were presented. It was shown that the proposed novelty detection algorithm was able to detect novelties in both kinds of data (real and synthetic) and that the proposed approach using simple adaptive models should be suitable for adaptive novelty detection. The detection rate of the proposed algorithm was evaluated for various SNRs in the scenarios of trend change detection and of a step change in the parameters of a signal generator. These scenarios were also tested with LE, ELBND, and prediction error evaluation. It was shown that for higher SNRs, the proposed ESE algorithm outperformed the other tested algorithms in terms of a successful detection rate in both scenarios.

## Abbreviations

The following abbreviations are used in this manuscript:

ND	novelty detection
ESE	extreme seeking entropy
LE	learning entropy
ELBND	error and learning based novelty detection
GEV	generalized extreme value
GPD	generalized Pareto distribution
GNGD	generalized normalized gradient descent
NLMS	normalized least mean squares
pdf	probability density function
cdf	cumulative density function
POT	peak over threshold
LNU	linear neural unit
QNU	quadratic neural unit
SNR	signal-to-noise ratio

## Appendix A

**Table A1:** Trend change detection rates.

SNR (dB)	ESE (%)	ELBND (%)	LE (%)	Err (%)
41.63	99.0	68.1	98.9	54.8
35.62	98.4	68.3	98.4	54.2
29.61	97.3	70.2	97.3	56.9
26.11	96.1	74.6	95.7	59.3
23.64	94.3	74.0	94.1	61.5
21.74	92.9	74.9	92.7	62.1
20.20	91.6	74.0	91.4	62.1
18.92	90.4	74.2	89.4	63.9
17.81	89.4	73.4	89.1	65.8
16.85	88.8	72.6	88.0	65.8
16.00	87.5	74.5	87.0	66.7
15.23	86.5	74.4	85.5	67.3
14.55	85.5	74.0	84.0	68.2
13.92	84.5	75.3	83.8	70.4
13.35	84.2	75.3	83.2	69.9
12.82	83.6	75.6	82.4	70.9
12.34	82.6	76.7	81.2	72.1
11.88	81.6	76.2	80.4	72.8
11.46	80.9	75.6	79.3	72.0
11.07	79.8	75.2	78.4	71.8
10.70	78.7	74.7	77.3	72.4
10.02	76.9	75.0	75.6	73.0
9.42	75.6	74.2	74.0	72.3
8.88	73.9	72.8	71.3	72.3
8.39	72.2	72.1	69.1	71.9
7.95	70.4	70.4	66.9	70.3
7.54	69.1	68.3	65.2	68.8
7.16	66.5	67.1	63.0	67.7
6.82	64.4	66.7	60.7	66.7
6.20	59.2	63.2	56.1	63.9
5.67	55.4	58.6	52.1	60.6
4.99	49.0	52.5	46.2	55.6

**Table A2:** Step change detection rates for inputs drawn from uniform distribution U(−1,1).

SNR (dB)	ESE (%)	ELBND (%)	LE (%)	Err (%)
39.70	100.0	94.6	99.5	95.2
33.68	100.0	94.6	98.7	95.3
30.16	99.8	94.7	98.1	95.4
27.67	99.5	94.7	96.7	95.3
25.73	99.2	94.6	96.4	95.4
25.07	99.1	94.6	96.3	95.4
24.15	98.9	94.5	96.2	95.2
23.33	98.6	94.3	95.9	95.0
22.82	98.5	94.2	95.7	94.9
22.11	98.1	94.2	95.7	95.1
21.67	97.8	94.3	95.2	94.8
20.66	97.4	94.0	94.8	94.5
19.75	96.9	93.9	93.9	94.2
18.93	96.7	93.8	93.6	94.2
18.19	96.4	93.5	93.1	94.0
17.77	95.7	93.1	92.5	93.6
17.51	95.3	92.7	92.2	93.2
17.12	94.8	92.1	91.6	92.7
16.88	94.5	92.1	91.1	92.4
16.52	94.0	91.8	90.7	92.1
16.29	94.1	91.6	90.3	92.1
15.96	93.5	91.5	90.2	91.7
15.75	92.8	91.4	89.5	91.2
15.24	91.7	91.0	88.4	91.0
15.04	91.4	90.8	88.0	90.8
14.76	90.7	90.0	87.5	90.6
14.48	90.3	89.5	86.9	90.3
14.31	90.2	89.2	86.3	90.1
14.13	90.1	88.9	85.6	89.9
13.88	89.1	88.4	85.5	89.5
10.60	78.8	80.2	75.2	82.3
10.09	76.6	77.9	73.6	80.4
9.62	74.6	75.9	71.8	78.0
9.19	72.1	73.6	69.6	75.6
8.78	69.9	71.6	68.0	73.6
8.41	68.2	70.4	65.7	71.2
8.05	66.5	68.8	64.2	69.1
7.72	63.9	67.4	62.0	67.2
7.40	61.5	64.2	59.6	64.8
3.64	30.1	32.4	30.1	31.4
1.33	11.7	11.2	11.5	9.7

**Table A3:** Step change detection rates for inputs drawn from normal distribution N(0,1).

SNR (dB)	ESE (%)	ELBND (%)	LE (%)	Err (%)
43.86	100.0	96.1	99.9	94.8
37.84	100.0	96.3	99.2	94.8
34.32	100.0	96.1	98.4	94.9
31.83	99.8	95.8	98.1	94.7
29.89	99.6	96.0	97.7	94.6
28.31	99.4	96.0	97.1	94.6
26.97	99.3	95.7	96.9	94.4
25.82	99.1	95.7	96.9	94.5
24.80	98.9	95.7	96.4	93.9
23.89	98.5	95.6	96.1	93.6
23.06	98.3	95.6	95.9	93.2
22.31	97.9	95.0	95.5	92.7
21.96	97.6	94.8	95.3	92.5
21.62	97.5	94.7	95.3	92.4
20.98	96.5	94.2	94.5	91.6
20.39	96.1	94.1	93.9	91.5
19.84	95.8	93.5	93.8	91.2
19.32	95.6	93.1	93.3	90.6
18.83	95.2	92.7	92.6	90.0
18.37	94.9	92.5	92.3	89.7
17.93	94.7	92.2	91.8	88.5
17.52	94.3	91.8	91.6	87.5
17.12	93.4	91.0	90.4	87.1
16.39	92.1	89.9	89.4	86.1
15.71	90.1	88.6	87.8	84.6
15.09	88.6	87.1	86.8	83.6
14.52	87.3	86.4	85.8	82.0
13.98	85.0	84.9	84.2	79.5
13.73	84.3	83.9	83.7	78.9
13.48	83.6	83.3	82.7	78.1
13.02	82.1	81.7	81.6	76.4
12.16	79.7	78.9	78.8	73.6
11.77	78.6	77.8	77.9	72.3
11.40	77.6	76.1	76.0	70.5
11.05	75.9	74.8	74.1	69.3
10.88	75.0	74.1	73.6	68.8
10.55	73.3	72.4	71.9	67.2
10.09	71.3	70.4	69.7	65.5
9.67	68.8	68.7	67.0	63.9
9.27	67.1	65.9	65.0	62.2
9.01	65.8	64.0	63.5	60.9
8.77	64.2	62.0	62.0	59.0
8.54	62.4	60.2	60.7	57.0
8.10	59.9	58.1	57.8	54.9
7.89	58.2	56.8	56.4	54.1
7.41	55.5	55.1	53.8	50.5
6.97	52.1	52.2	50.8	46.6
6.57	49.0	49.6	48.6	44.2
6.20	45.9	47.3	45.6	42.1
5.54	41.0	40.7	40.1	37.8
4.98	36.3	36.5	36.5	34.7
4.50	33.0	32.4	32.3	30.6
4.08	30.0	28.8	28.4	27.3
3.71	27.4	26.0	25.9	24.5
3.39	24.2	24.1	23.9	22.3
3.10	21.6	22.2	21.4	20.5
2.85	19.9	20.3	19.2	19.4
2.63	18.5	18.1	17.5	17.8
2.43	16.9	16.5	16.3	16.6
2.25	15.8	15.4	15.3	15.2
2.09	14.7	14.3	14.0	14.0
